# Toward robust surgical phase recognition via deep ensemble learning

**DOI:** 10.1007/s11548-025-03543-6

**Published:** 2025-11-08

**Authors:** Flakë Bajraktari, Lina Hauser, Peter P. Pott

**Affiliations:** Institute of Medical Device Technology, University of Stuttgart, Pfaffenwaldring 9, 70569 Stuttgart, Germany

**Keywords:** Phase recognition, Deep ensemble learning, Model diversity, Surgical assistance

## Abstract

**Purpose:**

Automatic recognition of surgical workflows is a complex yet essential task of context-aware systems in the operating room. However, achieving high accuracy in phase recognition remains a challenge due to the complexity of surgical procedures. While recent deep learning models have made significant progress, individual models often exhibit limitations—some may excel at capturing spatial features, while others are better at modeling temporal dependencies or handling class imbalance.

**Methods:**

This study investigates the use of ensemble learning to combine the complementary strengths of diverse architectures, aiming to mitigate individual model weaknesses and improve performance in surgical phase recognition using the Cholec80 dataset. A variety of advanced deep learning architectures was integrated into a single ensemble. Models were carefully selected and tuned to ensure diversity, resulting in a final set of 15 unique ensembles. Ensemble strategies were explored to determine the most effective method for combining the distinct models.

**Results:**

The results demonstrated that ensemble learning significantly improved performance. Among the ensemble strategies tested, majority voting achieved the highest F1-score, followed by the proposed artificial neural network StackingNet. Ensembles with high model diversity showed superior performance compared to those with lower diversity. The optimal ensemble configuration integrated top-performing models from different architectures, leading to improvements in accuracy, F1-score, and Jaccard Index by 1.48 %, 3.68 %, and 5.43 %, respectively, compared to the best individual models.

**Conclusion:**

This study demonstrates that ensemble learning can substantially enhance surgical phase recognition by leveraging the complementary strengths of diverse deep learning models. Ensemble size, diversity, and meta-model selection were identified as key factors influencing performance. The resulting improvements translate into clinically meaningful benefits by enabling more reliable context-aware guidance, reducing misclassifications during critical phases, and improving surgeons’ trust in artificial intelligence (AI) systems.

## Introduction

**Fig. 1:**
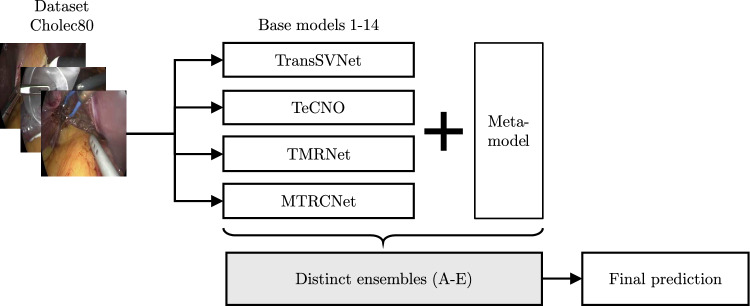
A schematic representation of the proposed process and components, which consists of four base models and their variations, accounting for a total of 14 models, all trained on Cholec80. One of 12 meta-models was applied to combine the models in each ensemble, resulting in 15 distinct ensembles (A–E) that ultimately produced the surgical phase predictions

The digital transformation of the operating room (OR) has become a focal point in contemporary medical research, with artificial intelligence (AI) playing a pivotal role in improving surgical workflows. Among the most impactful applications of AI in surgery is automated surgical phase recognition (SPR), which enhances intraoperative guidance and reduces the workload of OR personnel by automating tasks such as procedural documentation. Ultimately, this will lead to improved efficiency and patient safety [1–3].

In recent years, breakthroughs in machine learning, particularly in deep learning, have propelled the automation of SPR using datasets such as Cholec80 [3], a benchmark for SPR research. Sophisticated algorithms have demonstrated promising results in the automatic classification of surgical phases. Convolutional neural networks (CNNs), such as ResNet [4], serve as the backbone for many SPR architectures [3, 5–8]. Before passing individual frames to temporal models, spatial features are extracted through CNNs. Temporal models, including recurrent neural networks (RNNs), temporal convolutional networks (TCNs), and vision transformers (ViTs), model temporal dependencies in sequential data for SPR. RNNs process sequences step by step with memory cells to retain past information [9, 10], TCNs employ dilated convolutions to capture long-range dependencies efficiently [11–13], and Transformers leverage self-attention to analyze all time steps simultaneously [5, 14]. While these models have significantly advanced SPR, their effectiveness is often constrained by several challenges, including limited data availability due to privacy concerns and the difficulty of obtaining large-scale sets of annotated surgical videos [15]. Additionally, the variability in surgical techniques introduces challenges in model generalization, making it difficult to achieve consistent performance not only across different procedures and datasets but also between different models trained under varying conditions [16]. To address these challenges, ensemble learning offers a promising approach by combining multiple models to enhance prediction accuracy and robustness [17, 18]. By leveraging the strengths of different deep learning architectures, models can improve their prediction accuracy in image classification tasks such as cancer [18] and tool detection during surgery [19, 20]. This approach enhances consistency in predictions, making models more reliable in real-world surgical settings [21].

Although ensemble learning is a well-established strategy in machine learning, its potential in SPR remains underexplored. Most existing approaches rely on individual deep learning architectures, with limited investigation into ensemble methods that could exploit the complementary strengths of multiple models. This study addresses this gap by systematically applying and evaluating a range of ensemble techniques for SPR. While ensemble learning itself is not new, the proposed approach introduces domain-specific methodological innovations by combining architectures with complementary temporal and spatial modeling capabilities through a diversity-driven selection. The primary objectives were to evaluate different deep learning architectures on the Cholec80 dataset, compare the effectiveness of various meta-models, and analyze the impact of ensemble learning on phase recognition accuracy. This work aims to advance the development of more accurate and reliable AI-driven assistance systems in surgery.

## Methods

The framework of this study followed a stacking ensemble approach as shown in Fig. 1, integrating state-of-the-art base models for SPR, which are trained on Cholec80, and combining their class probability vectors through various meta-models, ultimately classifying the surgical phases.

### Dataset and base models

The Cholec80 dataset, a widely used benchmark for SPR, proposed by Twinanda et al. [3], was used for training and evaluation. It consists of a total of 80 videos of laparoscopic cholecystectomy procedures performed by 13 different surgeons. The recordings were captured at a frame rate of 25 frames per second (fps) with a resolution of either $1920 \times 1080$ or $854 \times 480$ pixels. Each video is annotated with seven distinct surgical phases, each varying in duration across different videos. For model training, the frame rate was reduced to 1 fps, and images were resized to $250 \times 250$ pixels. Additionally, data augmentation was applied using basic transformations, including cropping, random horizontal flipping, and random color variations. The data was split into a ratio of 32:8:40 for training, validation, and testing, respectively. Four different state-of-the-art models were identified as base models. To evaluate the ensemble with models of the same architecture, two RNN models with long short-term memory (LSTM) cells were included. Otherwise, all models differ fundamentally in their model architecture. The set consisted of a TCN (*TeCNO* [11]), a transformer-based model (*Trans-SVNet* [5]), and two LSTM-based models (*MTRCNet* [10] and *TMRNet* [22]). The models were trained and tested individually in cases where no pre-trained models were available or they did not adhere to the 32:8:40 data split. Otherwise, the hyperparameters from the original studies were applied. The Trans-SVNet model was trained to optimize it using a 32:8:40 data split, differing from the original study. Since the model incorporates a TeCNO component, the pre-trained TeCNO models used in this study were adopted for the TeCNO evaluation. Similarly, the MTRCNet model was trained, as tool detection for SPR was not required, and only the phase recognition branch of the model was utilized. For TMRNet, the hyperparameters from the original study were retained, as the model was already trained on Cholec80 using the defined data split, eliminating the need for additional hyperparameter optimization.

Each model was trained in three independent runs, and the reported performance metrics were computed through the mean across these runs. For the final evaluation, the epoch with the highest validation accuracy was selected. Additionally, various model variants were trained to increase diversity within the ensemble. The backbones ResNet50 [4] and ResNeSt50 [23] were used, and the number of feature maps and the number of multistage TCN (MSTCN) layers in *TeCNO* and *Trans-SVNet* were varied. This resulted in 14 different models. Training was carried out on a workstation ( Core$^{\textrm{TM}}$ i9-12900K, 5.2 GHz, HP Inc., CA, USA) equipped with a GeForce RTX 3080 Ti graphics card (NVIDIA Corp., CA, USA), running Ubuntu 20.04.01 (Focal Fossa). The PyTorch machine learning framework was used for model development, while Weights  & Biases (W&B) [24] was employed for hyperparameter tuning.

The F1-score was used for metric comparison, as the Cholec80 dataset is imbalanced, making the F1-score a more suitable measure for evaluation. In line with established evaluation protocols for surgical workflow recognition [25], the metrics were computed without relaxed boundaries, meaning that no tolerance window was considered around phase transitions to account for minor temporal deviations in annotations.

### Meta-models and ensembles

To combine the predictions of the base models, the following meta-models were used in this study:

- Majority voting (MV)
- Logistic regression (LR)
- Stochastic gradient descent (SGD)
- Random forest (RF)
- Decision tree (DT)
- AdaBoost (AB)
- Bagging classifier SVM (BSVM)
- Bagging classifier SGD (BSGD)
- Bagging classifier LR (BLR)
- Gaussian naïve Bayes (GNB)
- K-nearest neighbor (KNN)
- StackingNet (SN)

**Table 1:** Summary of ensembles implemented and evaluated in this study

Ensemble	Models	Description
A1	1, 2, 3, 4	All *Trans-SVNet* models
A2	5, 6, 7, 8, 9, 10	All *TeCNO* models
A3	11, 12	All *TMRNet* models
A4	13, 14	All *MTRCNet* models
B1	1, 2, 5, 6, 7, 11, 13	All models based on ResNet50 backbone
B2	3, 4, 8, 9, 10, 12, 14	All models based on ResNeSt50 backbone
C1	2, 6, 12, 14	Models from each architecture with highest F1-score
C2	2, 6, 11, 13	Top models from each architecture using ResNet50
C3	3, 9, 12, 14	Top models from each architecture using ResNeSt50
C4	2, 3, 6, 9, 11, 12, 13, 14	Top models across all architectures and backbones
D1	1–10	All *Trans-SVNet* and *TeCNO* models
D2	11–14	All *TMRNet* and *MTRCNet* models
D3	1–4, 11, 12	All *Trans-SVNet* and *TMRNet* models
D4	5–10, 13, 14	All *TeCNO* and *MTRCNet* models
E	1–14	All 14 models

The meta-models employed in this study vary in complexity and decision mechanisms. To illustrate the diversity of approaches, the underlying functions of three representative models are presented: majority voting, logistic regression, and StackingNet. Other ensemble methods such as random forest, AdaBoost, and bagging classifiers follow standard implementations using *Scikit-learn* [26], and are therefore not detailed here.

**Majority**: When using majority voting, the class label that
**Voting**: receives the highest number of votes among all base models is selected. Formally, for a classification problem with class set $\mathcal {C}$, the final predicted label $\hat{y}$ is given by
  1 $$\begin{aligned} \hat{y} = \arg \max _{c \in \mathcal {C}} \sum _{i=1}^{M} \mathbb {1}(h_i(x) = c), \end{aligned}$$
  where $M$ denotes the number of base models, $h_i(x)$ is the class label predicted by the $i$th base model for input sample $x$, and $\mathbb {1}(\cdot )$ is the indicator function, which equals 1 if its argument is true and 0 otherwise. This method relies exclusively on the discrete predictions of the base models without considering confidence scores.
**Stacking**: The StackingNet meta-learner used in this study
**Net**: was previously introduced by Bajraktari et al. [27], where its architecture and training procedure are described in detail. StackingNet is a three-layer feedforward neural network with two hidden layers that takes as input the concatenated predicted class probabilities from all base models. Let $\textbf{z} \in \mathbb {R}^M$ denote this input vector. The network computes the output $\hat{y}$ through successive layers as follows:
  2 $$\begin{aligned} \begin{aligned} \textbf{h}^{(1)}&= \phi ^{(1)}\big (\textbf{W}^{(1)} \textbf{z} + \textbf{b}^{(1)}\big ), \\ \textbf{h}^{(2)}&= \phi ^{(2)}\big (\textbf{W}^{(2)} \textbf{h}^{(1)} + \textbf{b}^{(2)}\big ), \\ \hat{y}&= \phi ^{(3)}\big (\textbf{W}^{(3)} \textbf{h}^{(2)} + \textbf{b}^{(3)}\big ), \end{aligned} \end{aligned}$$
  where $\textbf{W}^{(l)}$ and $\textbf{b}^{(l)}$ are the weight matrices and bias vectors of layer $l$, $\phi ^{(l)}(\cdot )$ denotes the activation function at layer $l$, ReLU for hidden layers and softmax for the output layer, and $\textbf{h}^{(l)}$ represents the activations of the $l$th layer. The output $\hat{y}$ corresponds to the predicted class probabilities.
**Logistic**: In the meta-learning context, logistic regression
**Regression**: models the posterior probability of each class as a function of the concatenated predicted class probabilities from all base models. Let $\textbf{z} \in \mathbb {R}^M$ denote the feature vector formed by concatenating these predicted probabilities, where $M$ is the total number of probability values across all base models. For multi-class classification, the class posterior probabilities, i.e., the probability that the true label is $c$ after observing the aggregated outputs $\textbf{z}$ from the base models, are computed as
  3 $$\begin{aligned} P(y = c \mid \textbf{z}) = \frac{\exp (\textbf{w}_c^\top \textbf{z} + b_c)}{\sum _{k \in \mathcal {C}} \exp (\textbf{w}_k^\top \textbf{z} + b_k)}, \end{aligned}$$
  where $c \in \mathcal {C}$ is the class index, and $\textbf{w}_c \in \mathbb {R}^M$ and $b_c \in \mathbb {R}$ are the weight vector and bias term corresponding to class $c$. The summation index $k$ ranges over all classes in $\mathcal {C}$, ensuring the posterior probabilities sum to one. The final predicted label is then
  4 $$\begin{aligned} \hat{y} = \arg \max _{c \in \mathcal {C}} P(y = c \mid \textbf{z}). \end{aligned}$$

For each meta-model, hyperparameter optimization was performed using grid search. The performance of each ensemble was assessed using the optimal hyperparameter set that achieved the best performance on the validation data.

**Table 2:** Individual performance of each model

Nr.	Model	Backbone	Layer	Feature maps	Metrics in %				
					Accuracy	Precision	Recall	F1-score	Jaccard
1	Trans-SVNet	ResNet50	8	32	$87.66 \pm 7.43$	$83.89 \pm 9.75$	$83.51 \pm 8.10$	$80.91 \pm 7.24$	$70.81 \pm 10.08$
2			9	64	$88.62 \pm 8.54$	$85.69 \pm 8.08$	$85.19 \pm 5.76$	$83.06 \pm 7.33$	$73.88 \pm 9.59$
3		ResNeSt50	8	32	$89.41 \pm 7.71$	$\mathbf {87.26 \pm 6.80}$	$82.36 \pm 12.16$	$82.83 \pm 7.70$	$72.89 \pm 11.11$
4			9	64	$\mathbf {89.93 \pm 6.04}$	$86.73 \pm 9.86$	$81.37 \pm 12.68$	$82.78 \pm 7.79$	$71.74 \pm 12.73$
5	TeCNO	ResNet50	8	32	$86.38 \pm 8.31$	$80.76 \pm 10.49$	$84.33 \pm 7.39$	$79.40 \pm 7.34$	$69.08 \pm 9.80$
6			9	64	$87.50 \pm 8.73$	$82.79 \pm 10.28$	$\mathbf {87.18 \pm 4.02}$	$82.37 \pm 7.05$	$72.86 \pm 9.65$
7			10	64	$87.70 \pm 6.86$	$84.21 \pm 10.00$	$82.05 \pm 10.35$	$81.26 \pm 6.70$	$70.48 \pm 9.53$
8		ResNeSt50	8	32	$87.53 \pm 8.29$	$82.82 \pm 10.16$	$81.70 \pm 13.03$	$79.40 \pm 9.47$	$68.93 \pm 12.08$
9			9	64	$89.28 \pm 6.21$	$84.56 \pm 10.90$	$84.15 \pm 7.09$	$82.31 \pm 7.71$	$72.74 \pm 10.88$
10			10	64	$89.08 \pm 7.36$	$84.52 \pm 10.20$	$83.78 \pm 8.55$	$82.21 \pm 7.62$	$72.38 \pm 10.76$
11	TMRNet	ResNet50	–	–	$87.52 \pm 9.61$	$84.59 \pm 5.60$	$84.42 \pm 6.29$	$82.25 \pm 6.37$	$72.50 \pm 8.41$
12		ResNeSt50	–	–	$88.35 \pm 8.37$	$85.04 \pm 5.97$	$85.43 \pm 5.64$	$\mathbf {83.59 \pm 5.61}$	$\mathbf {73.89 \pm 8.63}$
13	MTRCNet	ResNet50	–	–	$85.11 \pm 8.05$	$79.05 \pm 8.85$	$82.04 \pm 6.64$	$78.17 \pm 8.35$	$66.83 \pm 10.70$
14		ResNeSt50	–	–	$85.76 \pm 7.87$	$79.66 \pm 8.20$	$83.35 \pm 6.43$	$79.23 \pm 7.55$	$68.21 \pm 9.71$

Best values for each metric are highlighted

For the quantitative evaluation of the ensemble models, the F1-score of the best-performing base model within each combination was selected as the reference. Subsequently, the performance improvements achieved by the meta-models over the best individual model in each configuration group A–E were calculated, providing relative performance gains.

The ensemble configurations listed in Table 1 were designed to address key research questions regarding the effectiveness of ensemble learning for SPR as detailed below. The model numbers and their corresponding model architectures can be extracted from Table 2.

- **Model architecture-based ensemble (A1–A4):** Evaluation of whether ensembles, composed of variations of the same architecture, achieve higher performance compared to individual models.
- **Backbone-based ensembles (B1–B2):** Evaluation of whether ensembles, grouped by backbone architecture, lead to different classification performances.
- **Performance-based ensembles (C1–C4):** Evaluation of whether ensembles, built from the best-performing models based on the F1-score ranking, outperform ensembles that include all available models.
- **Heterogeneous model architecture in ensembles (D1–D4):** Evaluation of both homogeneous (D2) and heterogeneous (D1, D3, D4) ensembles to assess the effectiveness of combining similar models versus mixing different architectures.
- **Full ensemble (E):** Evaluation of the performance of a single ensemble containing all available models.

## Results

The best-performing models based on the evaluation metrics are summarized in Table 2. The highest observed accuracy is 89.93 %, while the maximum precision, recall, F1-score, and Jaccard Index are 87.26 %, 87.18 %, 83.59 %, and 73.89 %, respectively.

**Fig. 2:**
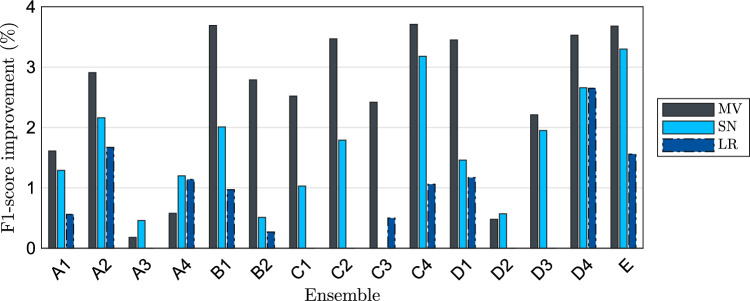
F1-score improvements of each ensemble including the three best-performing meta-models, majority Vote (dark gray), StackingNet (light blue), and logistic regression (dark blue), relative to the best-performing base model within the respective ensemble

The ensemble performances were assessed across all meta-models. Figure 2 illustrates the F1-score improvements in  % for the meta-models relative to the best-performing model within its respective ensemble. For better visibility, only the three best-performing meta-models majority voting, StackingNet, and logistic regression are displayed.

Majority voting emerged as the most consistently high-performing meta-model, achieving the highest rank in 12 out of 15 configurations, including a 3.71 % improvement over the baseline in configuration C4. It also ranked second in two configurations and third in one. StackingNet followed, attaining the top rank in three configurations and second-highest performance in 11. Logistic regression showed competitive results, ranking second once and third in seven configurations. These three meta-models consistently demonstrated superior performance compared to the others, indicating their effectiveness in the ensemble learning framework for surgical phase recognition.

## Discussion and outlook

The evaluation of the ensemble models reveals key insights into the impact of model diversity, backbone architectures, and meta-model selection on classification performance. The results indicate that ensembles consisting of multiple variations of the same network architecture can enhance classification performance compared to individual models. This effect is particularly evident in ensembles A1 and A2, where the use of different configurations of ResNet50 and ResNeSt50 led to improvements of 1.61 % and 2.91 %, respectively, with majority voting as the meta-model. The increased diversity in model configurations appears to contribute to higher performance, as the ensemble can capture a wider range of feature representations. In contrast, ensembles with only two networks, as in A3 and A4, show more modest improvements, with the best results achieved using StackingNet (0.46 % for A3 and 1.2 % for A4). This suggests that while integrating different backbone architectures within an ensemble can lead to performance gains, the limited number of networks may restrict the potential benefits. This pattern is further supported by D2, where only one architecture type is used. This combination also fails to show substantial performance improvements, reinforcing the idea that architectural diversity within an ensemble is a key factor for achieving higher performance.

The analysis reveals that ensemble C4 with majority voting achieves the highest F1-score of 87.27 %, making it the best-performing ensemble in this study. This ensemble achieves improvements, with particularly notable increases in F1-score (+3.68 %) and Jaccard Index (+5.43 %). The rise in Jaccard Index suggests that the ensemble improves the alignment between predicted and actual classes, leading to more precise classification. Interestingly, C1, which consists of the best-performing model from each architecture, does not surpass the performance of C4. This indicates that a diverse ensemble does not automatically yield the best results; rather, having a sufficient number of complementary models appears to be the key factor. The ability of the ensemble to learn from both strong and weaker individual models reinforces the benefits of aggregating multiple perspectives in the classification process. This pattern is supported by the marginal difference between C4 (87.27 %) and E (87.24 %), which suggests that ensemble performance improves primarily with the number of networks included, rather than solely relying on selecting the best individual models. This finding emphasizes that quantity, alongside quality, plays a crucial role in ensemble effectiveness.

Regarding meta-model performance, majority voting emerges as the most effective approach, ranking first in 12 of the 15 ensembles regarding F1-score improvements. StackingNet follows closely, ranking second in 11 cases. A key observation is that the effectiveness of majority voting increases with the number of models in the ensemble. This aligns with the idea that a larger ensemble benefits from greater model diversity, which helps mitigate biases and capture a broader spectrum of patterns in the data. The combination of multiple models with different configurations allows majority voting to leverage the strengths of each, leading to more robust predictions. Conversely, in smaller ensembles, where base models may generate highly similar predictions, a learned meta-model like StackingNet is better suited to identify nuanced decision patterns.

From a clinical perspective, improved phase recognition accuracy has tangible implications for intraoperative assistance systems. In particular, ensembles with high diversity reduce misclassifications during critical phase transitions, thereby supporting reliable context-aware decision-making. Even improvements of a few percentage points, which may appear modest in absolute terms, can substantially lower error rates in real surgical procedures. Clinically, this translates into more consistent workflow predictions, enhanced trust in AI-driven systems, and improved safety during surgery. For clinical deployment, the ensemble must also remain effective over time. A dynamic ensemble management framework could be implemented in which the ensemble configuration is updated regularly. In the process, performance metrics such as F1-score are automatically monitored using a continuously updated validation set derived from recent surgeries. Based on these metrics and pre-defined thresholds, the ensemble is then reassembled by selecting the top-performing models, while outdated or underperforming models are removed during regular software updates. Newly trained or fine-tuned models are integrated in the latest version, ensuring that only the best-performing models in the optimal combination remain active. This dynamic mechanism would enable continuous improvement of ensemble performance in clinical use.

While ensembles improve performance, it is important to note that they also increase computational and energy demands compared to single models. This trade-off highlights the need to balance accuracy gains with efficiency considerations, particularly for clinical applications where real-time deployment and sustainability are crucial.

Another aspect worth reflecting on is the choice of strict evaluation boundaries. Relaxed boundaries typically yield higher metric values, since small temporal shifts around phase transitions are tolerated. However, this can also blur the interpretation of how well a model truly captures phase boundaries. In line with recent findings in the field, a strict evaluation protocol was chosen. While this may result in more conservative performance values, it ensures that reported improvements more directly reflect genuine advances in recognition rather than artifacts of the evaluation setup.

The selection of ensemble strategies in this study was not intended to exhaust all possible combinations of the 14 base models, which would have resulted in an unmanageable number of ensembles. Instead, ensembles A–E were designed to systematically address specific research questions. This targeted approach enabled a structured evaluation while keeping the study scope tractable. Nevertheless, the design space of ensemble methods in SPR remains broad. Future work could extend the analysis to a wider range of combinations or incorporate alternative paradigms to further investigate their potential benefits. One possibility is to apply boosting strategies at the base model level, where models are trained sequentially and each model focuses on the errors of previous models. Such approaches could increase complementarity between base learners and potentially lead to more powerful ensembles. Another promising direction is Bayesian aggregation at the meta-model level. Instead of combining base model outputs through fixed or globally learned rules, Bayesian aggregation combines them probabilistically and infers weights that reflect each model’s confidence and reliability. This could improve robustness, particularly in uncertain or rare surgical situations.

Overall, the results demonstrate that ensembles effectively enhance classification performance, with model diversity, ensemble size, and meta-model selection being key factors influencing the outcome. While larger ensembles tend to perform better with statistical methods like majority voting, ensembles with fewer models may benefit more from meta-models that can learn complex relationships between predictions. The findings of this study provide a strong foundation for further research in SPR. Expanding the ensemble to include a wider range of architectures and model combinations could further increase diversity and capture complementary information, which may lead to higher robustness and accuracy. From a clinical perspective, such improvements translate into more reliable intraoperative assistance, fewer misclassifications during critical phases, and greater consistency in decision support. Ultimately, these developments could enhance surgeon trust in AI systems and contribute to improved patient safety in computer-assisted surgery.

## Data Availability

The dataset used is publicly available. No new data were generated in this study.
